# Percutaneous Closure Device Controlled INCRAFT Stentgraft Implantation Registry (PUCCINI)

**DOI:** 10.1186/s42155-025-00523-4

**Published:** 2025-02-08

**Authors:** T. Engelen, R. Hoogervorst, K. DeLoose, L. C. van Dijk, R. S. van Eps, R. B. van Tongeren, H. T. Veger, L. Maene, W. Stomp, O. R. Wikkeling, S. J. C. Klink, W. van den Eynde, J. J. Wever, H. van Overhagen

**Affiliations:** 1 Interventional Radiology, Haga Teaching Hospital, The Hague, The Netherlands; 2 Department of Vascular Surgery, AZ St. Blasius, Dendermonde, Belgium; 3 Department of Vascular Surgery, Haga Teaching Hospital, The Hague, The Netherlands; 4 Department of Vascular Surgery, Deventer Hospital, Deventer, The Netherlands; 5 Department of Vascular and Thoracic Surgery, OLV Aalst, Aalst, Belgium; 6 Department of Vascular Surgery, Nij Smellinghe, Drachten, The Netherlands; 7 Department of Vascular and Thoracic Surgery, Imelda, Bonheiden, Belgium

**Keywords:** Percutaneous endovascular repair (PEVAR), Infra-renal abdominal aortic aneurysm, Suture-mediated closure, Low-profile endograft

## Abstract

**Background:**

Percutaneous endovascular repair (PEVAR) of infra-renal abdominal aortic aneurysms (AAA) is increasingly being performed due to the development of low profile endografts and the use of percutaneous closure devices. The feasibility and safety of the use of the INCRAFT AAA Stentgraft System and the ProGlide vascular closure system was assessed.

**Methods:**

The PUCCINI trial prospectively enrolled patients undergoing elective repair of infrarenal AAA at 3 centres in the Netherlands and 3 centres in Belgium. Patients underwent PEVAR with endograft implantation followed by closure using the ProGlide closure device. Procedural success rates, complications and 30-day follow-up outcomes were collected.

**Results:**

A total of 93 patients, 87% male, were enrolled. The mean aneurysmal diameter was 53.9 ± 10.2 mm. Successful ProGlide placement was achieved in 97.2% in the right and 89.8% in the left groin. Successful closure was achieved in 92.4% of right and 90% of left groins. One patient required surgical access and two surgical closure. Average blood loss was 155.6 ± 175.5ml. Blood transfusion was not required. Average length of hospital stay was 2.1 ± 1.3 days. Post-implantation endoleaks were present in 37 (40.2%) patients (type 1: 12, type 2: 25). At 30-days there was no aneurysmal growth and no deaths. Follow-up imaging showed endoleaks in 39 (41.9%) patients. (type 1:8, type 2:29, type 3:2).

**Conclusion:**

The results from the PUCCINI trial demonstrate that the use of a low profile endoprosthesis for treatment of infrarenal AAA with percutaneous closure has a high rate of technical success and low rates of periprocedural complication.

## Introduction

Endovascular aortic repair (EVAR) has become the standard treatment for infrarenal abdominal aortic aneurysms (AAA). Initially, stentgrafts used for EVAR were bulky and rigid, necessitating surgical cutdown in the groin for vascular access, which was associated with local groin complications such as wound infections [1–4].

Innovations in stent material, fabric, and material constraints have constantly led to the development of low-profile devices [5–7]. In 2009 reported sheath size for stentgrafts was 21–24 French [8] whereas nowadays devices with sheath size of 14 French are available [5]. A key benefit of these smaller stentgrafts is the possibility of percutaneous implantation using closure devices, eliminating the need for surgical cutdown (percutaneous EVAR (PEVAR)). Despite the increasing popularity of these percutaneous closure devices, the technique has not yet become a widespread standard procedure and there are only limited published results of prospective studies.

The aim of this study is to prospectively collect technical and clinical performance data to evaluate the efficacy and safety of PEVAR using the small sized INCRAFT stent-graft system, since results of PEVAR have been reported to improve with small sized devices [9] and access managed with the ProGlide percutaneous closure device system for electively treating infrarenal AAA.

## Materials and methods

### Study design

The PUCCINI trial was an investigator initiated, prospective multi-centre, post-market, non-randomized single-arm registry, that describes elective percutaneous treatment of patients with an abdominal aneurysm with the INCRAFT stentgraft (Cordis, Florida Lakes, USA) and the ProGlide closure device (Abbott, Chicago, USA). It was conducted at 3 centres in the Netherlands and 3 centres in Belgium. It is descriptive in nature, collecting data of patients, procedures and follow up. Post-operative follow-up adhered to the standard care protocol after EVAR, with assessments scheduled at 1 month and 1 year post treatment. Data were collected in a prospective matter by using Castor Electronic Data Capture. Syntactx (NAMSA) was assigned as central research organisation (CRO) and for data management. The initial goal was to include 100 consecutive patients. This seemed to be a reasonable number for analysis. However, due to the COVID pandemic, inclusion lagged behind and for reasons of funding we were forced to set a time line to inclusion. Thus, inclusion ran from October 2019 till August 2023. Because of the nature of the study results are given in absolute numbers and percentages without comparing statistics. The study protocol was approved by the Medical Ethics Review Committee South-West Holland and the study was registered at the Netherlands Trial Register under trial ID NL-8044. The PUCCINI investigators received a non-restricted study grant from Cordis and Abbott. The primary efficacy endpoint of the study was technical success, defined as successful percutaneous access and closure with the ProGlide closing device for EVAR with the INCRAFT stentgraft. Successful closure was defined as complete haemostasis obtained with the ProGlide closing device and a maximum of 5 min post placement manual pressure. Primary and secondary safety endpoints of the study, as well as the clinical utility measures, can be found in Table 1.

**Table 1:** Primary endpoints, secondary endpoints and clinical utility measures

Primary endpoints	Secondary endpoints	Clinical utility measures
Absence of surgical conversion at the access sit	Number of percutaneous closure device failure	procedural time (from beginning of (local) anaesthesia to removal of surgical drapes)
Blood transfusion due to bleeding at the access site	number of surgical conversions at the access site	fluoroscopy time
Absence of procedure related major events at one month	Number of patients with local anaesthesia, sedation, spinal and general anaesthesia	contrast volume used
Major adverse events • All-cause mortality • Aneurysm rupture related mortality • Stentgraft related mortality • Myocardial infarction • Bowel ischemia • Stroke • Renal ischemia causing dialysis • Surgical conversion at the aneurysm site • Surgical conversion at the access site	periprocedural blood loss	number of closure devices used
		number of ICU days
		number of days in hospital

### Patients

Eligible subjects had to be at least 18 years old and had to give informed consent prior to the procedure. All patients underwent computed tomography angiography (CTA) within 3 months prior to treatment. Lab measurements (Creatinine, Glomerular filtration rate (GFR), haemoglobin (HB), IWBC and platelets) and body measurements (height, weight, body mass index (BMI), blood pressure) were collected. All subjects had to be eligible for both open and endovascular AAA repair.

#### Inclusion criteria

The AAA had to meet one of the following criteria: a diameter of ≥ 50 mm for women and ≥ 55 mm for men, a demonstrated growth of ≥ 5 mm within the past 6 months, or the presence of a saccular AAA. Treatment had to be elective in nature, thus only non-ruptured AAAs could be included.

**Fig. 1:**
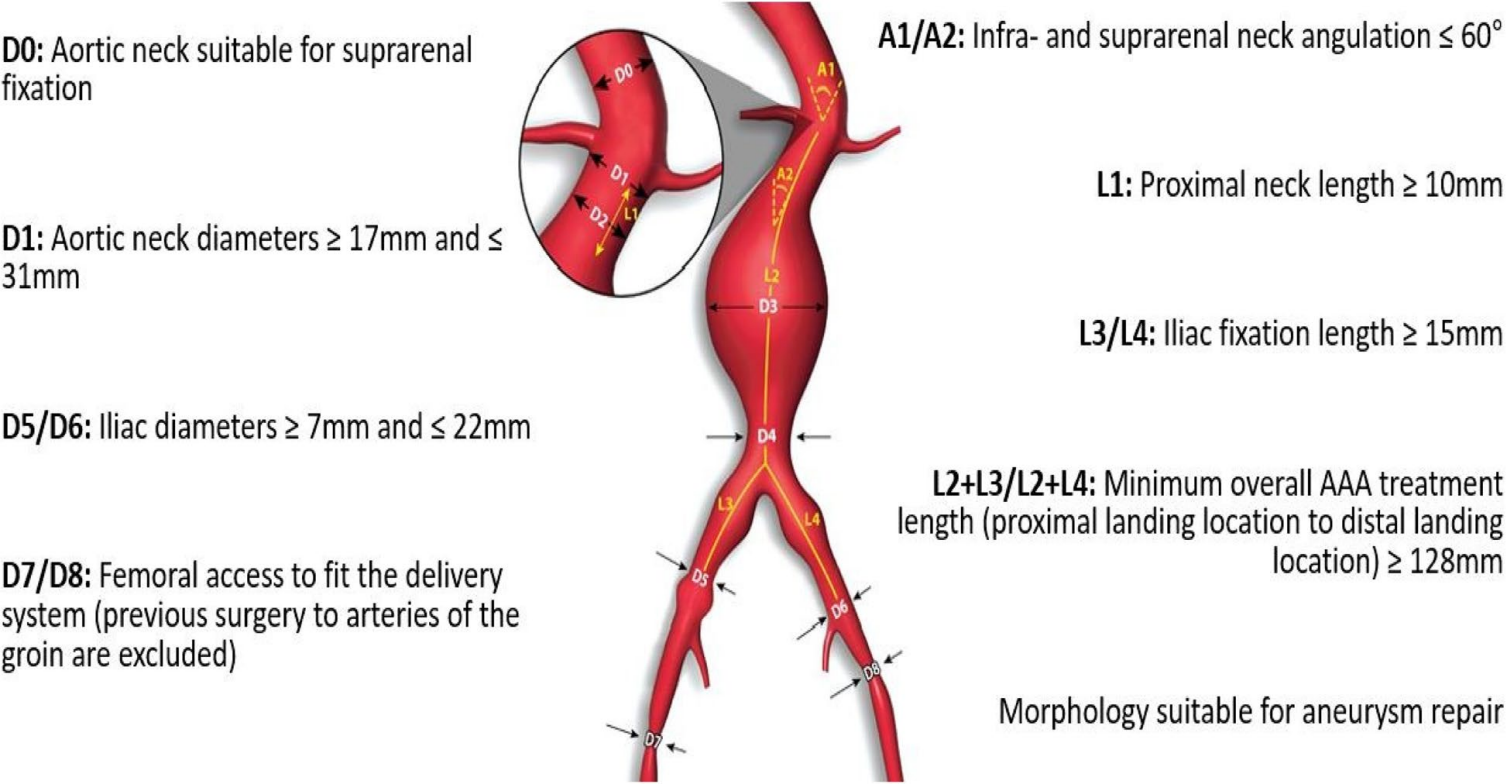
Inclusion criteria by vessel and abdominal aortic aneurysm characteristics

Further inclusion criteria were according to instructions for use (IFU) and are shown in Fig. 1.

#### Exclusion criteria

Exclusion criteria are shown in Table 2.

**Table 2:** Exclusion criteria

Dissection of the infra-renal Aorta
Ruptured Aorta
Infra-renal Aorta Angulation > 60°
Aortic Length (lowest renal artery to aortic bifurcation) < 8.7cm
Substantial Thrombosis and/or calcification in the infra-renal neck
Glomerular Filtration Rate (GFR) < 20
High probability of non-adherence to the study protocol
Incapability to communicate with the patient
Existing AAA device in situ
Mycotic aneurysm
Present or planned pregnancy during the course of the trial
Previous surgery of arteries of the groin
Critical lower limb ischemia

### Materials

#### Closure device

The Perclose ProGlide™ Suture-Mediated Closure System by Abbot medical is a percutaneous suture that is introduced through the femoral puncture site to close the femoral artery and prevent bleeding [10, 11]. The stitch is made of a polypropylene material that will keep the femoral artery closed and will not dissolve over time. The system can be used with or without adjunctive manual compression.

For access sites in the common femoral artery, the system is suitable for sheath sizes ranging from 5 to 21 French. When using arterial sheaths larger than 8 French, a minimum of two devices and the pre-close technique, introduction before insertion of the stentgraft, are required. In this trial, as the sheaths used were larger than 8 French, a minimum of two closure devices per groin was recommended [11, 12].

#### Stentgraft

The INCRAFT AAA stentgraft system is a tri-modular device, consisting of one aortic bifurcate main body prosthesis and two iliac limb prostheses. The endograft is made of a seamless, low-porosity woven polyester graft, supported by short, electropolished, self-expandable Nitinol stent rings along its entire length. These endoskeletal stents are laser-cut from a low-profile Nitinol tube and sutured to the inner surface of the graft material. Radiopaque markers are attached to the device to aid in fluoroscopic positioning. The aortic bifurcate prosthesis includes a short infrarenal sealing endoskeleton and a proximal trans renal bare stent with laser-cut barbs at the cranial apex of each bare stent [13].

The system incorporates a customizable tri-modular design. Its main body is available in sizes ranging from 22 up to 34 mm diameter, with an ipsilateral length of 94 mm and contralateral length of 86mm.The iliac legs are available in diameters ranging from 10 to 24 mm diameter with lengths ranging from 82 to 138 mm. The delivery system has a 14 French outer diameter for the main body and 12 French for the legs [14].

### Methods

#### Access

Patients were sedated in combination with local anaesthesia, or underwent general anaesthesia consistent with local practice and investigator choice at each centre. Arterial access, bilaterally, was recommended by using ultrasound guidance. Subsequently two ProGlides were typically placed in each groin for each patient prior to endoprosthesis implantation. For the placement of the ProGlides a number of parameters were recorded which were ease of access, number of ProGlides used, number of failures and(un)successful placement of the sutures.

#### Implantation

The main body of the INCRAFT Stent-Graft was inserted according to the instructions for use (IFU) [13] and placement was checked using digital subtraction angiography (DSA) and x-ray imaging. Hereafter typically the ipsilateral leg of the graft was placed in position, followed by the contralateral leg.

After placement and checking positions, each graft was balloon moulded to secure the graft in its desired location. Angiography was subsequently performed to evaluate graft function and endoleaks. Data which were noted included: insertion and placement of the stentgraft, size of graft used, endo leaks, complications, (un)planned over stenting of arteries, successful removal of device, additional procedures (extension, stenting) and balloon moulding.

### Closure

After confirming successful placement, the closure of the groins was performed using the already placed ProGlides. Necessity and duration of manual pressure was noted. Up to 5 min of manual pressure was considered as within normal limits. In addition, the need for surgical cut down was documented.

### Additional data

Further data collected were total procedure time, total fluoroscopy time, amount of contrast material used, blood loss, length of hospital stay and ICU stay. Follow up was planned for 1 month and 1 year after implantation, including CTA or duplex and laboratory examinations.

This trial was conducted in three centres in The Netherlands (Haga Teaching Hospital, Den Haag, Deventer Hospital, Deventer and Nij Smellinghe Hospital, Drachten) and three centres in Belgium (OLV Aalst, Imelda Hospital Bonheiden, AZ St. Blasius Hospital Dendermonde).

The Medical Ethics Review Committee (METC) South-West Holland declared this study ‘non-WMO-obligatory research'. This advice was followed by the Belgian ethics committees.

## Results

### Inclusion

A total of 93 patients were included during the study period, 81 male and 12 female, aged 58–94 (mean 73 ± 7,2) years, and a BMI from 18–41 (mean 26,3 ± 4,49) (Table 3).

**Table 3:** Baseline characteristics of participants in the study. Values shown as Mean (SD), when applicable

	**Number**		
**Patients**	***N* = 93**		
Male	*N* = 81		
Female	*N* = 12		
Age	73(7.2)		
**Medical history**			
Coronary artery disease	*N* = 25	Peripheral artery disease	*N* = 10
Myocardial infarct	*N* = 14	Claudication	*N* = 5
CABG	*N* = 12	Critical limb ischemia	*N* = 0
Hypertension	*N* = 49	Diabetes Type 2	*N* = 10
Congestive heart failure	*N* = 19	Cholesterol ≥ 8 mmol/l	*N* = 29
TIA	*N* = 3	(former) Smoker	*N* = 67
Stroke	*N* = 4	Major relevant illness	*N* = 13
Carotid endarterectomy/Stent	*N* = 3		
**Lab measurements**			
BMI	26.3(4.49)		
GFR ml/min	69.8(16.5)		
Creatinin μmol/L	91.7(26.1)		
Leukocytes × 109/L	7.37(2.36)		
Platelets × 109/L	221(91.4)		
Haemoglobin mmol/L	9.78(4.5)		

### CTA measurements

CTA measurements can be found in Table 4.

**Table 4:** Characteristics of vascular anatomy

**Aneurysm**			
Max. AP diameter		53.9 (10.2)	
Max. diameter		58.6 (8.9)	
**Aorta**			
Diameter 20 mm. prox. to distal renal artery		24.6 (3.5)	
Diameter at distal renal artery		22.9 (3.2)	
Diameter 10mm. below distal renal artery		22.9 (2.9)	
Infrarenal neck length		31.6 (13.5)	
Infrarenal neck angulation (°)		27.3 (21.4)	
Diameter distal aorta		25.3 (7.0)	
Max. diameter aortic bifurcation		28.9 (10.9)	
Min. diameter aortic bifurcation		22.9 (8.5)	
**Treatment length**			
Lowest renal to aorta bifurcation		121 (21.2)	
Aortic bifurcation to iliac bifurcation right		68.9 (19.9)	
Aortic bifurcation to iliac bifurcation left		66.6 (17.7)	
**Iliac access right**			
Max. diameter common iliac artery		15.5 (4.5)	
Diameter at hypogastric take-off		13.6 (3.2)	
External iliac diameter at access		8.0 (2.5)	
Calcification right ICA		Thrombus right ICA	
0–90°	*N* = 70	0–90°	*N* = 79
91–180°	*N* = 17	91–180°	*N* = 10
181–270°	*N* = 5	181–270°	*N* = 0
271–360°	*N* = 0	271–360°	*N* = 0
**Iliac access left**			
Max. diameter common iliac artery		14.9 (3.9)	
Diam. at hypogastric take-off		13.4 (3.1)	
External iliac diameter at access		8.1 (2.6)	
Calcification left ICA		Thrombus left ICA	
0–90°	*N* = 57	0–90°	*N* = 76
91–180°	*N* = 26	91–180°	*N* = 12
181–270°	*N* = 8	181–270°	*N* = 2
271–360°	*N* = 0	271–360°	*N* = 0

Values shown as mean (SD), in Millimetre(mm), AP: anteroposterior

Infrarenal neck configuration was cylindrical in 64, conical in 15, hourglass shaped in 9, barrel shaped in 2, and tapered in one patient, respectively. Not recorded in the 2 final patients.

### Procedure

Forty of 92 (43.5%) patients had general anaesthesia, 36 (39.1%) sedation with local anaesthesia, 10 regional anaesthesia and 6 local anaesthesia only.

#### Access

Ninety-two of 93 included patients underwent EVAR. One patient was eventually not treated according to the trial protocol due to non-availability of the stentgraft. Successful access to the right groin was achieved in 98% (91/92) of all patients. One patient required open access. A total of 183 ProGlides were placed, of which 5 failed, leading to a success rate of 97,2%. Reasons for failure included breaking of the sutures (*N* = 4) and preliminary tying of the knot (*N* = 1).

Successful access to the left groin was achieved in 100% (92) of all patients. No open access was required. A total of 177 ProGlides were placed, of which 18 failed, leading to a success rate of 89,8%. Reasons for failure included breaking of sutures (*N* = 4) device failure (*N* = 1) and failures without reasons given (*N* = 13).

Thus, the primary efficacy endpoint for successful access was 98–100%.

#### Implantation

Implantation of the stentgraft was done according to the IFU and was successful in all cases. In 84 of 92 (91,3%) patients, the main device was inserted through the right groin (84/92). At final angiography an endoleak was seen in 37 (40,2%) patients. In 25 patients this was a type 2 endoleak (26,8%), in 11 patients a type 1A endoleak (11,8%) and in one patient a type 1B endoleak (1%).

An additional procedure was performed in 1 patient before insertion of the main device. This was a PTA of the right iliac artery. In 11 patients additional procedures were performed after placement of the main device, including an infrarenal cuff (*N* = 1), PTA of the iliac leg (*N* = 3), stenting of the iliac leg (*N* = 5) and surgical cut down (*N* = 2).

In 11 cases there was intentional over stenting of arteries; renal artery (*N* = 3), accessory renal artery (*N* = 5) and internal iliac artery (*N* = 3). In one patient an internal iliac artery was unintentionally covered.

#### Closure

Right side closure was successful in 86 of 92 (92,4%) patients, with 5 patients requiring manual compression for more than 5 min (7–25 (mean 12 ± 6,6) minutes), and 1 patient requiring surgical cut down.

Left side closure was successful in 83 of 92 (90%) patients, with 7 patients requiring manual compression 5–20 (mean 8,9 ± 5,1) minutes, one patient requiring surgical cut down, and 1 unknown failure.

Thus, the primary efficacy endpoint for successful closure was 90–92%.

#### Additional data

Mean procedure time was 86 ± 27,3 (range 27- 169) minutes, from the initial incision to the final completion angiography. Average blood loss was 155,6 ± 175,5 (range 0 -1000) ml. None of the patients required blood transfusion, and there were no reported deaths. Fluoroscopy time ranged from 1.3–53 (mean 18,9 ± 9,1) minutes. Amount of contrast used was 25–250 (mean 105 ± 42) ml. Average hospital stay was 2,13 ± 1,28, (range 1 -7) days, with 0–1 (mean 0,09 ± 0,29) days of ICU stay. All data are shown in Table 5.

**Table 5:** (Peri)Procedural outcomes

**Anaesthesia**			Number (%)
General anaesthesia			40 (43.5%)
Sedation + local anaesthesia			36(39.1%)
Regional anaesthesia			10(10,9%)
Local anaesthesia only			6(6,5%)
**Access left**		**Access Right**	
Successful access	92(100%)	Successful access	91(98%)
Open access necessary	0	Open access necessary	1(1,1%)
ProGlides used	177	ProGlides used	183
ProGlide failures	18(10,2%)	ProGlide failures	5(2,8%)
**Implantation**			
Main device introduction right side			84(91,3%)
Main device introduction left side			8(8,7%)
**Additional procedures**			
**Before implantation**			1(1%)
PTA right iliac artery			1(1%)
**After implantation**			11(12%)
Infrarenal Cuff			1(1%)
PTA iliac leg			3(3,2%)
Stenting iliac leg			5(5,4%)
Surgical cutdown			2(2,17%)
**Intentional overstenting**			11(11,9%)
Renal artery			3(3,2%)
Accessory renal artery			5(5,4%)
Internal iliac artery			3(3,2%)
**Unintentional overstenting**			1(1%)
internal iliac artery			1(1%)
**Endo leak on final Angiography**			37(40,2%)
Type 1A			11(11,8%)
Type 1B			1(1%)
Type 2			25(26,8%)
**Closure**			
**Successful closure left side**	83(90%)	**Successful closure right side**	86(92,4%)
Manual compression > 5 min	7(7,6%)	Manual compression > 5 min	1(1%)
Surgical cutdown	1(1%)	Surgical cutdown	5(5,4%)
Unknown	1(1%)		
**Clinical utility measures**			**Mean (SD)**
Procedure time (min.)			86(27,3)
Blood loss (ml.)			155,6(175,5)
Fluoroscopy time (min.)			18,9(9,1)
Hospital stay (days)			2,13(1,28)
ICU stay (days)			0,09(0,29)

## 30 day follow up

All 92 patients were alive and underwent 30 day follow up with either CTA (*N* = 86) or duplex *N* = 22), and 16 patients underwent both. Average aneurysm diameter was 57 ± 7,0 (range 43 – 83) mm. No aneurysm growth was observed. Average GFR at follow up was 69,3 ± 18,2 ml/min.

An endo leak was assessed in 39 patients, Type 1 (*N* = 8), Type 2 (*N* = 29), Type 3 (*N* = 2).

Interval complications were assessed in 4 patients. One patient presented with a limb occlusion, one with a new renal artery occlusion on the right side, one patient presented with an internal iliac artery occlusion, and one had a false aneurysm of the common femoral artery. Thus, the primary safety endpoint, absence of surgical conversion, blood transfusion and major adverse events was met in 90 of 92 patients (96,7%).

## Discussion

This is the first study to report that elective PEVAR to treat aneurysms of the abdominal aorta using the INCRAFT stentgraft and ProGlide closure devices is an effective and safe procedure. Technical success rate of vascular access with the ProGlide system was 97–98% which is consistent with a recently published series, which showed a success rate of 95.4% with dual ProGlides [15], only once surgical access was necessary. This may have been facilitated by using ultrasound guided access. In our experience, vascular access may be hindered by vessel wall calcifications [16] and these can be seen and avoided by using ultrasound. Failure rate for closure with the ProGlide system was low (≤ 10%) and could be managed with prolonged manual pressure in the majority of cases despite reported suture breakage. Only in 2 patients surgical cut down was performed for closure. This is consistent with previous retrospective reports [12, 15, 16].Prolonged oozing after using the ProGlide system is encountered incidentally and may be limited by using small access diameter stentgrafts such as the INCRAFT with an outer diameter of 14 French for the main body and 12F for the iliac legs. Although PEVAR is also performed with 18 French outer size stentgrafts, the surface of such a system is more than 1.5 times the surface of a 14 French system. In our series blood loss was limited with no transfusions and technical success rates of percutaneous closure devices have been reported to be higher with smaller stentgraft diameters [9].

The INCRAFT stentgraft was successfully introduced and deployed in all patients. There was a relatively high type 1 endoleak rate of 13% which was reduced to 8.6% at 1 month follow up, consistent with previous reports regarding this device( 6,6%) [7]. This trend in an initially relatively high type 1 endoleak rate has been reported previously by Liang et al. [6] who also report a high type 4 endoleak rate which we did not observe. The relatively high percentage of endoleaks on post implantation angiography may be partially caused by the thin graft material used in order to facilitate a small outer stentgraft diameter. Type 2 endoleaks at 30 days follow up were comparable with earlier reports [17]. There was no aneurysm growth after this limited follow up.

The INCRAFT stentgraft is a trimodular system with a small outer diameter that facilitates introduction and passage through narrow and calcified arteries. Clinical results have been previously reported in elective patients [17] and in ruptured abdominal aneurysms [18]. Because of the limited outer diameter of the stentgraft, leg arteries are not occluded by the device during the procedure with improves patient comfort and cooperation. More or less half of the patients in our study had sedation or local anaesthesia only. Thus, the combination of the INCRAFT stentgraft with a percutaneous closure device may be considered in an interventional suite, in short stay and perhaps on an outpatient basis in a selected group of patients.

PEVAR has been reported to have a lower access site complication rate than open access [3, 4, 7].

In addition, at present, EVAR is standard of care elective treatment for most patients with an AAA. In the USA there is an increase in EVAR in patients with an AAA and a decrease in open surgery [19].With this trend, PEVAR with small sized devices may be beneficial for logistic reasons.

In conclusion, the results from the PUCCINI trial demonstrate that the use of a low profile endoprosthesis for treatment of infrarenal AAA with percutaneous closure has a high rate of technical success and low rates of periprocedural complication.

## Data Availability

The datasets used and/or analysed during the current study are available from the corresponding author on reasonable request.
